# Changes in Fixation Stability with Time during Binocular and Monocular Viewing in Maculopathy

**DOI:** 10.3390/vision2040040

**Published:** 2018-10-23

**Authors:** Saba Samet, Esther G. González, Mark S. Mandelcorn, Michael H. Brent, Luminita Tarita-Nistor

**Affiliations:** 1 Krembil Research Institute, Toronto Western Hospital, Toronto, ON M5T 2S8, Canada; 2 Faculty of Medicine, University of Toronto, ON M5S 1A8, Canada; 3 Department of Ophthalmology and Vision Sciences, University of Toronto, ON M5T 2S8, Canada

**Keywords:** central vision loss, age-related macular degeneration, fixation stability, worse eye, preferred retinal locus, fixation duration

## Abstract

The purpose of this study was to examine changes in fixation stability over time during binocular and monocular viewing in patients with age-related macular degeneration (AMD). Seventeen patients with AMD and 17 controls were enrolled. Using an EyeLink eyetracker (SR Research Ltd., Mississauga, Ontario, Canada), fixation stability was recorded binocularly and monocularly with each eye for a duration of 15 s while the fellow eye was covered. Fixation stability was analyzed over 3 s intervals for each condition using a 68% bivariate contour ellipse area. Fixation stability did not change with time during binocular viewing for both groups, both monocular conditions for the control group, and monocular viewing with the better eye for the AMD group. However, during monocular viewing with the worse eye, the test of within-subject contrasts showed linear improvement in fixation stability with time (*p* = 0.016). In conclusion, in patients with AMD, monocular fixational control with the worse eye is poor, but improves with time.

## 1. Introduction

Age-related macular degeneration (AMD) destroys central vision, but damages to the two eyes are often unequal, resulting in differences in monocular function. Binocular vision is primarily driven by the better eye (BE), but it could be affected negatively by a large dissimilarity in monocular function. Some patients with advanced AMD experience binocular inhibition of acuity and contrast sensitivity, where binocular performance is worse than monocular performance with the BE [1,2,3]. Patients with binocular inhibition show severe impairment in reading speed compared to those with central vision loss matched in visual acuity of the BE [4,5]. It appears that in these patients the worse eye (WE) has a heavy influence on binocular function by diminishing its performance.

In addition to the degree of central retinal damage, visual performance is related to the ability of the ocular motor system to adapt to the loss of its reference position (i.e., the fovea). Patients with AMD use preferred retinal loci (PRLs) in the functional eccentric retina as their pseudo-foveae, but the choice of PRL location is involuntary and the mechanisms not fully understood [6,7,8,9,10,11]. Both fixation stability and PRL location influence visual performance [12,13,14,15]. A great deal of research has been dedicated to studying monocular and binocular fixation stability as well as monocular PRL location [16,17,18]—technical limitations have prevented the study of the PRL location during binocular viewing in more detail. A rare paper of binocular PRL locations in patients with bilateral AMD has shown that, in a case with large differences in retinal damage for the two eyes, the PRL location of the BE did not change during monocular and binocular viewing; the PRL of the WE however was located in the far eccentric functional retina for monocular viewing, only to move and come in retinal correspondence with the PRL of the BE during binocular viewing [19]. This change resulted in a PRL location inside the scotoma (i.e., in the blind area) for the WE and this is likely the habitual location since binocular vision is predominantly used for daily visual function. Moreover, it has been shown that monocular fixation stability with the WE is poor, but improves during binocular viewing to the level of the BE’s, suggesting that the WE does not disrupt binocular ocular motor control [20]. These findings imply highly adaptable ocular motor control even with the WE.

For the well-established PRLs, fixation stability does not change over time during monocular viewing. That is, fixation stability is the same over consecutive time periods of equal length [21]. These findings are important because (1) the clinical tests for identifying the PRL and fixation stability with imaging instruments can be shortened and therefore can increase the patient’s comfort without compromising the results, and (2) they provide insight into the robustness of ocular motor control during fixation with central vision loss. While we expect similar results for binocular viewing, this may not be the case for monocular viewing with the WE, particularly for patients with large differences in visual function between the two eyes. In this study, using previously recorded data, we examined the changes in fixation stability over time during monocular and binocular viewing for patients with AMD and healthy controls. Data on fixation characteristics in the left eye are important considering the frequent use of the WE as the study eye in clinical trials, and the inhibitory effects it may have on binocular visual function.

## 2. Materials and Methods

### 2.1. Participants

Data from 17 patients with a confirmed diagnosis of AMD (9 males, 8 females, mean age 78.6 ± 8.1 years) and 17 participants with normal vision (8 males, 9 females, mean age 31.0 ± 13.7 years) were reviewed from our research database. All had participated in previous studies of fixation stability for which approval was obtained from the University Health Network Research Ethics Board (ethical approval code 08-1067-AE) [20]. None had coexisting ocular pathologies (with the exception of mild cataract), cognitive impairment, or a history of neurologic disease. Fifteen patients had bilateral AMD and 2 had only one eye affected. 

Visual acuity at 6m was assessed with a computerized version of the Early Treatment of Diabetic Retinopathy Study chart (single line) and a letter-by-letter scoring system was used. Mean binocular visual acuity was −0.11 ± 0.12 logMAR for the control group and 0.34 ± 0.22 logMAR for the AMD group. For the patients, monocular visual acuities were also assessed; the BE and the WE were identified as the eyes with the best and worst acuity, respectively. Two patients had equal visual acuities in the two eyes, and thus the eye with better fixation stability was selected as the BE. Mean visual acuity of the BE was 0.34 ± 0.20 logMAR and that of the WE was 0.93 ± 0.48 logMAR. All patients had an established PRL in the better eye, identified with the Nidek MP-1 microperimeter (Nidek Technologies Srl., Padova, Italy) during a monocular fixation task. We also examined the PRL in the worse eye, and this data has been reported by our group previously [20]. Clinical and demographic characteristics of the patients are shown in Table 1.

### 2.2. Apparatus and Procedure

Apparatus and Procedure have been previously described [20]. In short, eye positions during fixation were recorded with a desktop remote EyeLink 1000 eye-tracker (SR Research Ltd., Mississauga, ON, Canada) at a sampling rate of 250 Hz. Calibration was performed with binocular viewing by using a standard 5-point grid with a nine-cycle square-wave radial grating subtending 4.38° of visual angle [22]. This procedure was followed by verification and drift correction routines. The fixation target was a 3° red cross presented in the middle of a Samsung monitor (Sync Master 900 NF) at a viewing distance of 60 cm. The monitor had a white background with a useful field of view of 34.4 × 26 cm. To allow for quick cover of each eye, two large (10 cm × 10 cm) double IR filters (Kodak Wratten 87 IR) were mounted at eye level on a hinged mechanism in front of the headrest. This setup has been used successfully for previous studies conducted in our lab. The filter prevents fixation on the target for the covered eye, while permitting the eye-tracker to continue recording its position. There were no cases in which the filter did not cover the eye. Furthermore, the second monitor of the EyeLink eye tracker allows monitoring of each eye independently. 

Testing was done in a well-illuminated room while the participants sat with their head stabilized by a chin and forehead rest. They were instructed to keep their gaze steady on the middle of the fixation target. Horizontal and vertical eye positions of both eyes were recorded continuously in three viewing conditions: (1) binocular viewing only; (2) monocular right eye (OD) viewing, left eye (OS) covered; (3) monocular OS viewing, OD covered. Eye-tracker recordings for each condition were only initiated after the participant fixated on the target binocularly. Approximately 2 s after beginning a trial, the experimenter initiated recording of eye position for a duration of 15 s.

### 2.3. Data Analysis

Viewing conditions for the AMD group were (1) binocular viewing; (2) BE viewing/WE covered; (3) WE viewing/BE covered. Viewing conditions for controls were (1) binocular viewing; (2) OD viewing/OS covered; (3) OS viewing/OD covered. In order to analyze fixation stability changes over time, each 15-second period was divided into 3-second consecutive intervals (5 intervals). For each 3s time interval, fixation stability was quantified with a 68% bivariate contour ellipse area (BCEA) [23] for each eye during binocular and monocular viewing (fellow eye with no visual feedback but its data recorded). That is, 30 BCEA values were computed for each participant (i.e., 3 viewing conditions × 2 eyes × 5 time intervals). The BCEA calculation assumes that the data are normally distributed on the horizontal and vertical axes. Although this assumption is often violated, the BCEA has become one of the most common metric for quantifying fixation stability and has high correlation and agreement with the isoline method, which does not assume normality [16,17,24,25]. Far outliers in the raw data (±3SD) were removed to eliminate undue influence on the BCEA and a log_10_ transformation was applied to the BCEA values to normalize the data. Data were analyzed with repeated-measures ANOVAs, separately for each group, using a Greenhouse-Geisser correction and an alpha level of 0.05. Familywise error rate across the pairwise comparisons was controlled with the Bonferroni approach.

## 3. Results

### 3.1. Control Group

**Figure 1:**
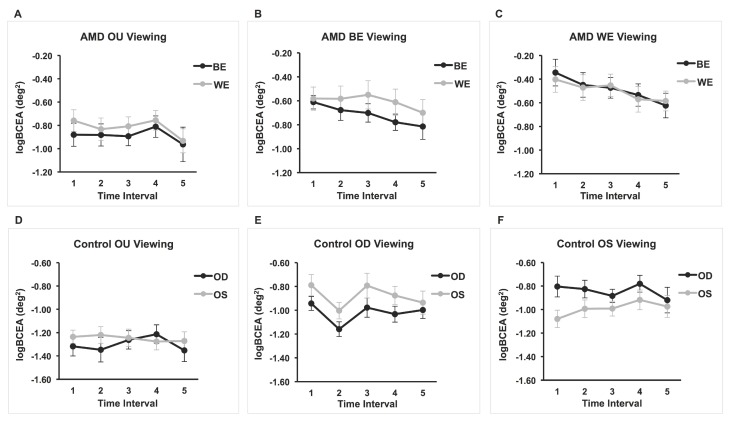
Mean logBCEA for five 3-second intervals for AMD and control groups under binocular and monocular viewing conditions. Fixation stability of both covered and viewing eyes are plotted for each viewing condition. (**A**) *n* = 17; (**B**) *n* = 16; (**C**) *n* = 15; (**D**) *n* = 17; (**E**) *n* = 16; (**F**) *n* = 17.

Three separate 2 × 5 repeated-measures ANOVAs were performed in order to examine the effect of eye (OS and OD) and time interval (every 3 s up to 15 s) on fixation stability (log_10_BCEA) during (1) binocular viewing, (2) monocular viewing with OS (OD covered), and (3) monocular viewing with the OD (OS covered). For the binocular viewing condition, there were no significant main or interaction effects (smallest *p* = 0.13). For the monocular viewing with the OS (OD covered), there was a significant main effect of eye, F(1,16) = 11.03, *p* = 0.004, partial η^2^ = 0.408. That is, the fixation stability of the viewing eye was significantly better than that of the covered eye. However, there was no significant main effect of time interval or interaction effect (smallest *p* = 0.20). Likewise, for the monocular viewing with the OD (OS covered), there was a significant main effect of eye F(1,15) = 7.037, *p* = 0.018, partial η^2^ = 0.319. For this condition too, the log_10_BCEA of the viewing eye was significantly better than that of the covered eye. There was no significant effect of time interval or interaction effect (smallest *p* = 0.068). Means and standard deviations are shown in Table 2 and the results are also displayed in Figure 1 (panel figure).

### 3.2. AMD Group

Three separate 2 × 5 repeated-measures ANOVAs were performed in order to examine the effect of eye (BE and WE) and time interval (every 3 s up to 15 s) on fixation stability (log_10_BCEA) during (1) binocular viewing, (2) monocular viewing with the BE (WE covered), and (3) monocular viewing with the WE (BE covered). For the binocular viewing, none of the main effects or interaction effects were significant (smallest *p* = 0.12). Likewise, there were no main or interaction effects for the BE viewing (smallest *p* = 0.082). Data for the BE (covered eye) during WE viewing of two patients were not reliable (i.e., too many data points missing or a lot of noise in the eye-movement recording) and were not included in the ANOVA. There were no significant interaction or main effects, although the time interval main effect approached significance (*p* = 0.055). The test of within-subject contrasts showed a significant linear effect of time, F(1, 14) = 7.47, *p* = 0.016, partial η^2^ = 0.35, suggesting that fixation stability improves linearly with time. Indeed, graphical representation of the results in Figure 1C shows an improvement in BCEAs for both the viewing eye and the covered eye with the passage of time. The average BCEA of the first interval was 1.52 larger than that of the last time interval for the WE. For the BE—the eye with no visual feedback—the control is poor but improves with each time interval; the average BCEA of the first interval was 1.90 larger than that of the last time interval. Means and standard deviations are shown in Table 3.

**Figure 2:**
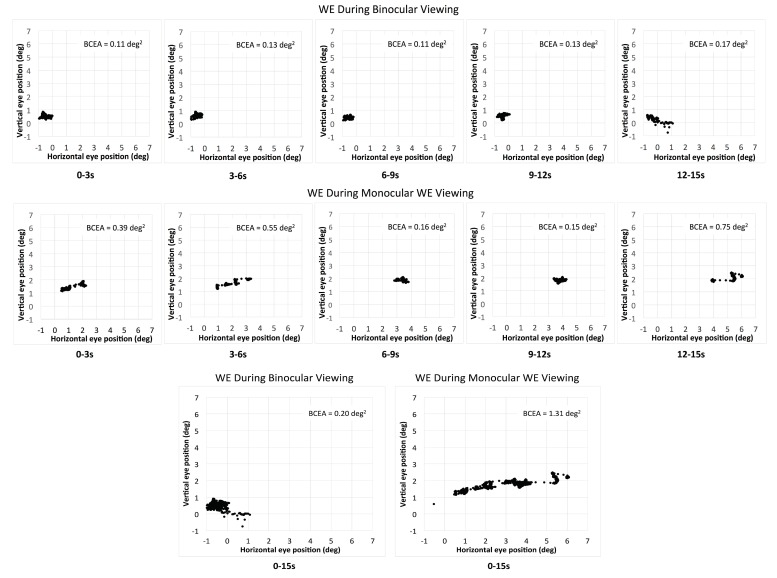
The eye position of the WE (worse eye) during binocular viewing (upper panel) and monocular WE viewing (middle panel) for the five consecutive intervals for patient P4. The bottom panel shows the total combined 15s interval for the two viewing conditions for the same patient.

**Figure 3:**
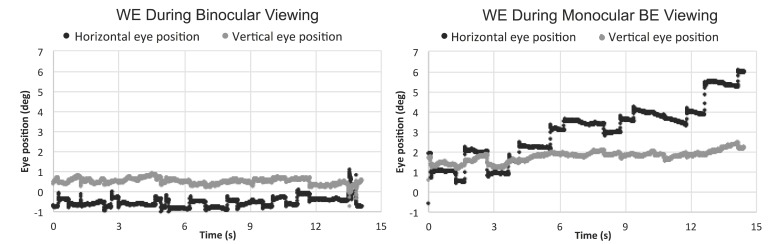
Horizontal and vertical eye position of the WE (worse eye) during binocular and monocular WE viewing for the 15s fixation period.

Eleven out of 17 patients had large interocular acuity differences (average interocular acuity difference of 0.88 logMAR, range 0.4 to 2 logMAR), suggesting substantial unequal damages to the two eyes. The existence or the search for a different PRL during monocular viewing compared to binocular viewing for the WE was probable. The relative change in the PRL location can be detected when there is a shift in the average of the horizontal and vertical eye position at each interval and viewing condition. For the patients with large interocular acuity difference, one patient (P3) showed evidence of a different PRL (relative change in horizontal eye position during monocular viewing with the WE for the five intervals was constant and ranged from 1.5 to 1.8 deg) and three others showed evidence of search for a new PRL location. The relative change in the horizontal eye position for the five intervals ranged from 1.3 to 5.1 deg for P4 (see example in Figure 2), 1.7 to 3.7 deg for patient P9, and 0.2 to 3.1 deg for patient P17. For the six patients with minimal interocular acuity differences, there were no changes in the PRL location: all values at any given interval were within 1 deg. Figure 3 shows the eye position traces of the WE for the 15s interval during binocular viewing and monocular viewing with the WE for patient P4.

## 4. Discussion

Using previously recorded eye-position data [20], we examined changes in fixation stability over time during monocular and binocular viewing in controls and in patients with AMD. We found that fixation stability does not change during consecutive time intervals of equal length during binocular and monocular viewing for controls and during binocular and monocular viewing with the BE for patients. However, during monocular viewing with the WE, fixation stability improves linearly with time.

It is important to study the WE because of at least two reasons. First, for clinical trials involving pharmacological therapies and rehabilitative measures, the WE is often chosen as the study eye, and it has been suggested that fixation stability should be used as an outcome measure after intervention [15]; while a moderate relationship between fixation stability and visual acuity has been repeatedly reported for the BE, none exists for the WE [20,26]. The large change in fixation stability even during a 15 s interval—fixation stability of the first 3 s interval is 1.52 times greater than that of the last interval—may explain this lack of correlation. Second, the WE can diminish binocular visual performance (i.e., binocular inhibition), but there is no evidence that it affects the ocular motor control during binocular viewing in a negative way. We have shown that fixation stability is poor during monocular viewing with the WE, but improves to the level of BE’s performance during binocular viewing [20]. These findings along with our current report that fixation does not change over time during binocular viewing bring further support that the WE does not affect the binocular ocular motor control during fixation.

The effects on fixation in patients with central vision loss has significant implications, one of many being reading. A study by Calabrèse et al., 2014 showed that reading speed is correlated with fixation duration, number of fixations, and with letters per forward saccade [27]. Fixation duration during reading is very brief even in patients with central vision loss—an average of about 280 ms [27]. For this reason, fixation duration and the number of fixations are more important measures rather than fixation stability when examining the influence of fixational ocular motor control on reading speed. However, a relationship between reading speed and fixation stability recorded during a fixation task of 10s (and not during reading) has been established: fixation instability is associated with poor reading speed in patients with central vision loss [14]. As our study has shown that the BE is the driver of binocular ocular motor control, and that fixation stability remains constant with time in this condition, rehabilitative measures directed to the BE can reliably improve daily visual function, including reading.

Why does fixation stability during monocular viewing with the WE improve with time? Two thirds of patients had large interocular acuity differences, which implies that one eye was more severely affected by a scotoma in the area of fixation, than the fellow eye. It may happen that during binocular viewing, the PRL in the WE falls on the scotoma to be in corresponding position with that of the BE, while during monocular viewing with the WE the patient uses a PRL in the functional peripheral retina. Although the new location of the PRL in the worse eye falls on the scotoma, the location of the PRL of the better eye remains unchanged, likely providing an advantage given that the PRL of the better eye dominates during binocular viewing. Technological limitations have prevented the study of the PRL location during binocular viewing concurrently for the two eyes to a great extent, but the scarce evidence suggests that this may be the case [19] (see Figure 4 in Tarita-Nistor et al., 2015). When changing the PRL location with viewing condition, patients may either know to use an established PRL or may search for a new PRL. In any case, it takes time for the ocular motor control with the WE to stabilize during a fixation task; our study shows that the control is poor for the first 3 s (first interval), but improves and stabilizes in 9 s of recording (first three intervals).

We have previously suggested that fixation examination with the MP-1 microperimeter could be shortened to a 5 s test, since fixation stability does not change with the passage of time [21]. The current study extends the recommendations to binocular viewing and monocular viewing with the BE recorded with an eye-tracker. For the WE however, fixation duration should depend on whether the goal of the examination is to determine fixation stability (i.e., variability of eye position during the task) or to determine the PRL location. For some patients—especially those with large interocular acuity differences—longer fixation examinations may reveal an inflated BCEA caused by a change in PRL location rather than by a large variability in eye position around a single PRL (see Figure 2). 

One of the limitations of our study is that results may have been influenced by age, as the mean age of the AMD group was about 48 years higher than the control group. Several ocular motor functions have been shown to be affected by age [28,29]. However, as it has been shown that age does not affect fixation stability [30,31], the results are more likely due to eye pathology.

In conclusion, in patients with AMD fixation stability stays constant over consecutive time intervals during binocular and monocular viewing with the BE. Monocular fixational control with the WE is poor, but improves over time. These findings suggest highly adaptive ocular motor control which, however, requires longer time to adjust to monocular viewing with the WE.

## Figures and Tables

**Table 1:** Demographic and clinical characteristics of AMD group.

ID	Age, y	Gender	Visual Acuity, logMAR		
			BE	WE	OU
1	83	M	0.55	1.3	0.46
2	73	M	0.36	0.48	0.38
3	81	M	0	0.86	0
4	82	M	0.14	0.90	0.14
5	84	M	0.42	0.78	0.48
6	85	M	0.51	0.60	0.55
7	84	F	0.34	0.57	0.30
8	60	M	0.44	0.44	0.36
9	73	M	0.30	1.1	0.53
10	78	F	0.22	0.34	0.16
11	87	F	0.64	2.0	0.74
12	84	F	0.42	2.0	0.42
13	70	F	0.66	0.66	0.62
14	87	F	0.26	0.90	0.06
15	79	M	0.04	1.1	0.14
16	63	F	0.04	1.1	0.06
17	84	F	0.36	0.76	0.34

**Table 2:** Fixation stability quantified by logBCEA (mean ± SD) for each eye during binocular and monocular viewing conditions for the control group.

		Viewing Condition					
Interval Number	Time Interval	Binocular		Monocular OD		Monocular OS	
		OD	OS	OD	OS	OD	OS
1	0–3 s	−1.32 ± 0.08	−1.24 ± 0.06	−0.94 ± 0.06	−0.79 ± 0.09	−0.80 ± 0.09	−1.08 ± 0.07
2	3–6 s	−1.35 ± 0.10	−1.22 ± 0.07	−1.15 ± 0.06	−1.00 ± 0.07	−0.83 ± 0.08	−0.99 ± 0.08
3	6–9 s	−1.26 ± 0.08	−1.24 ± 0.08	−0.98 ± 0.08	−0.79 ± 0.10	−0.88 ± 0.06	−0.99 ± 0.06
4	9–12 s	−1.21 ± 0.08	−1.28 ± 0.07	−1.03 ± 0.07	−0.88 ± 0.08	−0.78 ± 0.07	−0.92 ± 0.08
5	12–15 s	−1.35 ± 0.10	−1.27 ± 0.08	−1.00 ± 0.07	−0.94 ± 0.10	−0.92 ± 0.11	−0.97 ± 0.09

OU viewing *n* = 17, OD viewing *n* = 16, OS viewing *n* = 17.

**Table 3:** Fixation stability quantified by logBCEA (mean ± SD) for the BE (better eye) and WE (worse eye) during binocular and monocular viewing conditions for the AMD group.

		Viewing Condition					
Interval Number	Time Interval	Binocular		Monocular BE		Monocular WE	
		BE	WE	BE	WE	BE	WE
1	0–3 s	−0.88 ± 0.10	−0.76 ± 0.09	−0.61 ± 0.06	−0.58 ± 0.10	−0.34 ± 0.11	−0.40 ± 0.11
2	3–6 s	−0.88 ± 0.10	−0.83 ± 0.10	−0.68 ± 0.09	−0.58 ± 0.11	−0.45 ± 0.10	−0.47 ± 0.11
3	6–9 s	−0.89 ± 0.08	−0.81 ± 0.08	−0.70 ± 0.08	−0.55 ± 0.12	−0.47 ± 0.09	−0.45 ± 0.09
4	9–12 s	−0.81 ± 0.09	−0.76 ± 0.08	−0.78 ± 0.07	−0.61 ± 0.11	−0.53 ± 0.09	−0.57 ± 0.11
5	12–15 s	−0.96 ± 0.15	−0.93 ± 0.10	−0.81 ± 0.11	−0.70 ± 0.11	−0.62 ± 0.10	−0.58 ± 0.08

OU viewing *n* = 17, BE viewing *n* = 16, WE viewing *n* = 15.

## References

[1] Faubert J., Overbury O. (2000). Binocular vision in older people with adventitious visual impairment: Sometimes one eye is better than two. J. Am. Geriatr. Soc..

[2] Valberg A., Fosse P. (2002). Binocular contrast inhibition in subjects with age-related macular degeneration. J. Opt. Soc. Am. A Opt. Image Sci. Vis..

[3] Quillen D.A. (2001). Effect of unilateral exudative age-related macular degeneration on binocular visual function. Arch. Ophthalmol..

[4] Tarita-Nistor L., Brent M.H., Markowitz S.N., Steinbach M.J., González E.G. (2013). Maximum reading speed and binocular summation in patients with central vision loss. Can. J. Ophthalmol..

[5] Fosse P., Valberg A. (2001). Contrast sensitivity and reading in subjects with age-related macular degeneration. Vis. Impair. Res..

[6] White J.M., Bedell H.E. (1990). The oculomotor reference in humans with bilateral macular disease. Investig. Ophthalmol. Vis. Sci..

[7] Crossland M.D., Culham L.E., Kabanarou S.A., Rubin G.S. (2005). Preferred retinal locus development in patients with macular disease. Ophthalmology.

[8] Crossland M.D., Engel S.A., Legge G.E. (2011). The preferred retinal locus in macular disease: toward a consensus definition. Retina.

[9] Greenstein V.C., Santos R.A., Tsang S.H., Smith R.T., Barile G.R., Seiple W. (2008). Preferred retinal locus in macular disease: characteristics and clinical implications. Retina.

[10] Von Noorden G.K., Mackensen G. (1962). Phenomenology of eccentric fixation. Am. J. Ophthalmol..

[11] Cheung S.H., Legge G.E. (2005). Functional and cortical adaptations to central vision loss. Vis. Neurosci..

[12] Amore F.M., Fasciani R., Silvestri V., Crossland M.D., de Waure C., Cruciani F, Reibaldi A. (2013). Relationship between fixation stability measured with MP-1 and reading performance. Ophthalmic. Physiol. Opt..

[13] González E.G., Tarita-Nistor L., Mandelcorn E.D., Mandelcorn M., Steinbach M.J. (2011). Fixation control before and after treatment for neovascular age-related macular degeneration. Investig. Ophthalmol. Vis. Sci..

[14] Crossland M.D., Culham L.E., Rubin G.S. (2004). Fixation stability and reading speed in patients with newly developed macular disease. Ophthalmic. Physiol. Opt..

[15] Mandelcorn M.S., Podbielski D.W., Mandelcorn E.D. (2013). Fixation stability as a goal in the treatment of macular disease. Can. J. Ophthalmol..

[16] Timberlake G.T., Sharma M.K., Grose S.A., Gobert D.V., Gauch J.M., Maino J.H. (2005). Retinal location of the preferred retinal locus relative to the fovea in scanning laser ophthalmoscope images. Optom. Vis. Sci..

[17] Tarita-Nistor L., González E.G., Markowitz S.N., Steinbach M.J. (2008). Fixation characteristics of patients with macular degeneration recorded with the MP-1 microperimeter. Retina.

[18] Kabanarou S.A., Crossland M.D., Bellmann C., Rees A., Culham L.E., Rubin G.S. (2006). Gaze changes with binocular versus monocular viewing in age-related macular degeneration. Ophthalmology.

[19] Tarita-Nistor L., Eizenman M., Landon-Brace N., Markowitz S.N., Steinbach M.J., González E.G. (2015). Identifying absolute preferred retinal locations during binocular viewing. Optom. Vis. Sci..

[20] Tarita-Nistor L., Brent M.H., Steinbach M.J., González E.G. (2011). Fixation stability during binocular viewing in patients with age-related macular degeneration. Investig. Ophthalmol. Vis. Sci..

[21] Tarita-Nistor L., Gill I., González E.G., Steinbach M.J. (2017). Fixation Stability Recording: How Long for Eyes with Central Vision Loss?. Optom. Vis. Sci..

[22] González E.G., Teichman J., Lillakas L., Markowitz S.N., Steinbach M.J. (2006). Fixation stability using radial gratings in patients with age-related macular degeneration. Can. J. Ophthalmol..

[23] Steinman R.M. (1965). Effect of target size, luminance, and color on monocular fixation. J. Opt. Soc. Am..

[24] Castet E., Crossland M. (2012). Quantifying eye stability during a fixation task: a review of definitions and methods. Seeing Perceiving.

[25] Kumar G., Chung S.T. (2014). Characteristics of fixational eye movements in people with macular disease. Investig. Ophthalmol. Vis. Sci..

[26] Kisilevsky E., Tarita-Nistor L., González E.G., Mandelcorn M.S., Brent M.H., Markowitz S.N., Steinbach M.J. (2016). Characteristics of the preferred retinal loci of better and worse seeing eyes of patients with a central scotoma. Can. J. Ophthalmol..

[27] Calabrèse A., Bernard J.B., Faure G., Hoffart L., Castet E. (2014). Eye movements and reading speed in macular disease: the shrinking perceptual span hypothesis requires and is supported by a mediation analysis. Investig. Ophthalmol. Vis. Sci..

[28] Sharpe J.A., Sylvester T.O. (1978). Effect of aging on horizontal smooth pursuit. Investig. Ophthalmol. Vis. Sci..

[29] Irving E.L., Steinbach M.J., Lillakas L., Babu R.J., Hutchings N. (2006). Horizontal saccade dynamics across the human life span. Investig. Ophthalmol. Vis. Sci..

[30] Kosnik W., Fikre J., Sekuler R. (1986). Visual fixation stability in older adults. Investig. Ophthalmol. Vis. Sci..

[31] Kosnik W., Kline D., Fikre J., Sekuler R. (1987). Ocular fixation control as a function of age and exposure duration. Psychol. Aging.

